# Systematic review and meta-analysis comparing zoledronic acid administered at 12-week and 4-week intervals in patients with bone metastasis

**DOI:** 10.18632/oncotarget.19856

**Published:** 2017-08-03

**Authors:** Ling Cao, Yong-Jing Yang, Jian-Dong Diao, Xu-He Zhang, Yan-Ling Liu, Bo-Yu Wang, Zhi-Wen Li, Shi-Xin Liu

**Affiliations:** ^1^ Department of Radiation Oncology, Cancer Hospital of Jilin Province, Changchun 130012, People's Republic of China; ^2^ Department of Oncology, China-Japan Friendship Hospital Affiliated Jilin University, Changchun 130012, People's Republic of China; ^3^ Department of Anesthesiology, The First Hospital of Jilin University, Changchun, Jilin, 130021, People's Republic of China

**Keywords:** zoledronic acid, bone metastasis, dose interval, skeletal-related events, meta-analysis

## Abstract

Zoledronic acid is used to treat patients with bone metastasis, but the optimal dosing interval remains controversial. We therefore performed a systematic review and meta-analysis to compare the efficacy and safety of a 12-week interval of zoledronic acid with the standard 4-week interval. Three randomized controlled trials comprising 2650 patients were analyzed. Using a random-effects model, pooled risk ratios (RRs) and 95% confidence intervals (CIs) were calculated. No differences in the occurrence of skeletal-related events (SREs: RR = 0.98; 95% CI = 0.86–1.12; *P* = 0.80) or grade 3/4 adverse events (RR = 0.91; 95% CI = 0.69–1.20; *P* = 0.52) were observed between the 12-week and 4-week groups. The 12-week group tended to have lower incidences of osteonecrosis of the jaw [13 (0.98%) vs. 23 (1.73%)] and kidney dysfunction [21 (1.68%) vs. 31 (2.45%)] than the 4-week group, though the difference did not reach statistical significance (RR = 0.58, 95% CI: 0.30–1.12; *P* = 0.11); (RR = 0.67, 95% CI: 0.39–1.15, *P* = 0.15). These data show that zoledronic acid administered at 12-week intervals instead of 4-week intervals does not increase the risk of SREs, and may reduce the incidence of osteonecrosis of the jaw and kidney dysfunction. This suggests the 12-week interval with zoledronic acid may be an acceptable treatment option.

## INTRODUCTION

Bone is one of the most common metastatic sites for malignant tumors, especially for breast, prostate, and lung cancers [1]. Bone metastases can cause skeletal-related events (SREs), which are associated with severe pain, bone fractures, hypercalcemia, nerve compression, and deterioration in the quality of life [2, 3].

Zoledronic acid is a highly effective drug that inhibits osteoclast-mediated bone resorption, and is approximately 100–1000 times more potent than other bisphosphonates [4, 5]. Zoledronic acid has been approved for the treatment of patients with bone metastasis [6, 7] and tumor-induced hypercalcemia [8]. Because SREs can repeatedly occur during bone metastases, the clinical guidelines of the American Society of Clinical Oncology recommend that zoledronic acid should be taken indefinitely every 3–4 weeks unless there is deterioration in the general health of patients [9].

Zoledronic acid is well tolerated, but the long-term use can produce serious toxic effects, including osteonecrosis of the jaw, nephrotoxicity, and hypocalcemia [10]. Importantly, the optimal dosing interval remains controversial [11]. Conventionally, zoledronic acid is given every 3–4 weeks, but this dosing interval was derived empirically, rather than from comparative studies or compelling pharmacodynamics data [12]. Longer schedules of zoledronic acid for the treatment of osteoporosis and bone metastases have been proposed in preclinical and retrospective studies, and randomized controlled trials (RCTs) [11–15]. However, there have been differences in the enrolled patients and administration of zoledronic acid between different RCTs. A previous meta-analysis study has examined the dosing interval of bone-targeting agents [16]; however, to the best of our knowledge, no study has specifically addressed the optimal dosing interval of zoledronic acid. To determine the efficacy and safety of a 12-week regimen of zoledronic acid, we conducted a systematic review and meta-analysis on this subject.

## MATERIALS AND METHODS

### Study selection criteria

The systematic review and meta-analysis were performed according to the Preferred Reporting Items for Systematic Reviews and Meta-Analyses (PRISMA) statement [17]. RCTs with a parallel design were included; studies with a quasi-randomized, single-arm phase II or non-original, and non-randomized trials were excluded. Enrolled patients had histologically proven malignant tumors with at least one site of bone involvement, regardless of the previous use of bone-targeting agents.

The primary endpoint was SREs, which were defined as any pathological fracture, spinal cord compression, radiotherapy to the bone, surgery involving the bone, or hypocalcemia. The secondary endpoints were grade 3 or 4 adverse events, osteonecrosis of the jaw, and kidney dysfunction. The grade of adverse events was assessed according to the Common Terminology Criteria for Adverse Events (CTCAE; version 3.0) [18].

### Literature search

There were no limitations regarding the publication year, publication status, or language in the electronic search. We searched several databases, including Embase, PubMed, Cochrane Library, and Web of Science, until March 20, 2017. Either Emtree or MeSH terms were used throughout the search schemes. To identify potentially qualifying articles, abstracts from academic meetings were also included. In addition to searching for original papers, a review of references was conducted. The search strategy for PubMed is provided in Appendix 1.

### Data extraction and assessment of the risk of bias

The literature search was independently conducted, and its quality was tested by two investigators. The risk of bias in the included studies was evaluated according to the handbook of the Cochrane Collaboration for systematic reviews of interventions [19]. A third reviewer was responsible for addressing disagreements when they occurred. The studies were considered to have unclear, low, or high bias risk based on the evaluation of the general sequence allocation, allocation concealment, blinding of personnel and participants (performance bias), outcome evaluation blinding (detection bias), partial addressing of the data, presence of biases in the reports, and other bias sources that could influence the validity of the research.

### Statistical analysis

Statistical analysis was conducted using RevMan 5.3 software (Nordic Cochran Centre, Copenhagen, Denmark, 2014). The risk ratio (RR) and the 95% confidence intervals (CI) were calculated to evaluate the data. The I^2^ and chi^2^ tests were employed for determining the shared heterogeneity among the studies. In the absence of heterogeneity (*P* > 0.10, I^2^ < 50%), the analysis was performed using a fixed-effects model. Otherwise, a random-effects model was used. Three potential sources of heterogeneity, namely statistical, clinical, and methodological, were studied. The I^2^ approach was used to measure heterogeneity; > 50% was regarded as a high level of heterogeneity, 25%–50% as a moderate level, and < 25% as a low level. If excessive heterogeneity occurred, descriptive statistics was employed to conduct the meta-analysis.

## RESULTS

### Included studies

**Figure 1:**
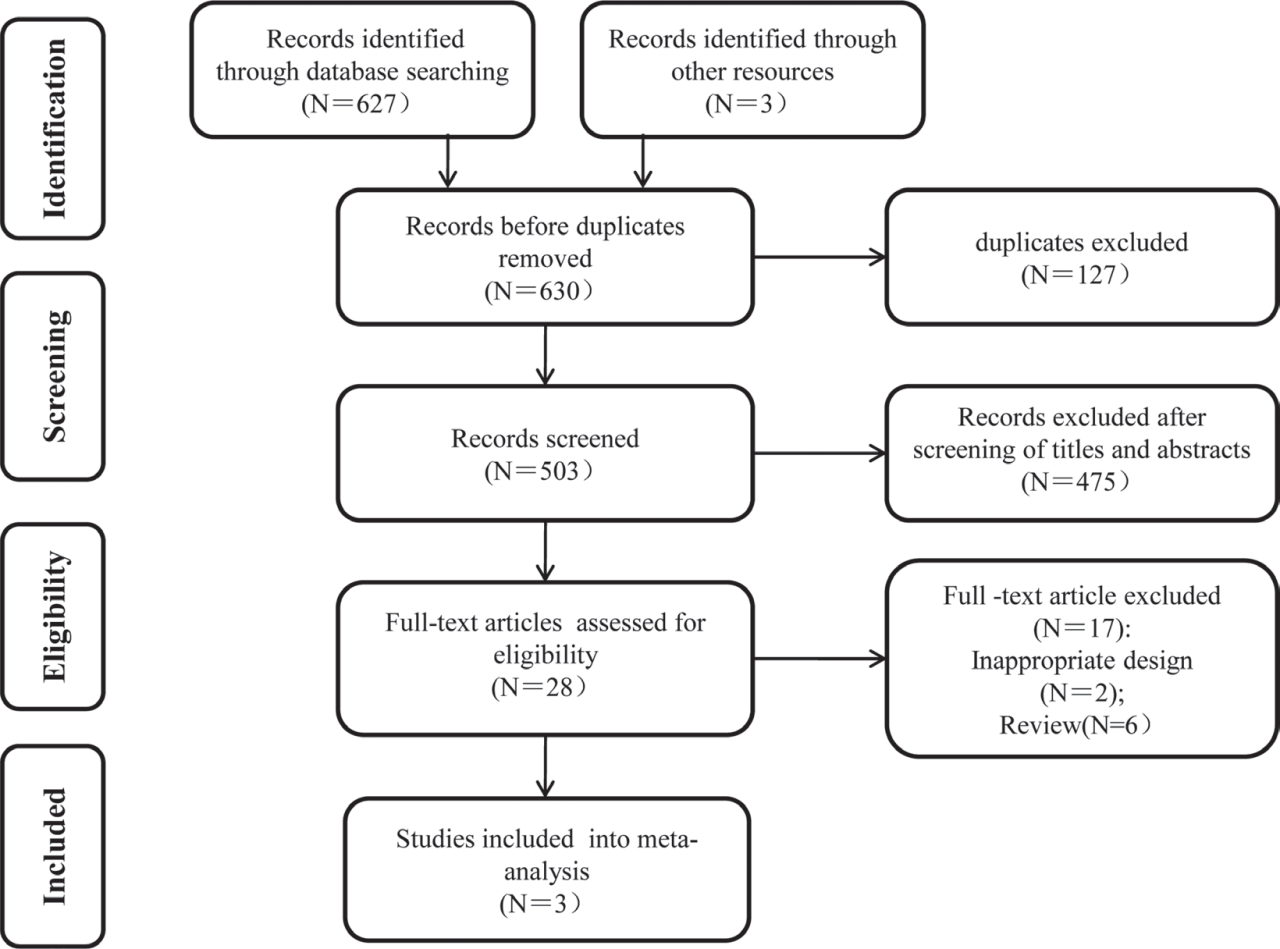
Flow chart of the study selection process

A total of 630 references were retrieved from the search, of which 127 were excluded as duplicates using the “find duplicates” feature of Endnote X7. Furthermore, 475 studies were excluded after the titles and abstracts were screened, and 28 full-text articles were selected for the evaluation of eligibility. Finally, three studies met the eligibility criteria (ZOOM 2013 [13], CALGB 70604 2017 [11], and OPTIMIZE-2 2017 [12]). Figure 1 shows the literature-screening process, and Table 1 lists the characteristics of the included studies. The meta-analysis comprised three RCTs with a total of 2650 patients.

**Table 1:** Baseline characteristics of the included trials

	ZOOM 2013 [13]	CALGB70604 2017 [11]	OPTIMIZE-2 2017 [12]
Enrolment time	Feb.2006–Feb.2010	May.2009–Apr.2012	Mar.2006–Jul.2013
Mean or median age (SD or range; years)	12w: 60.4 (11.9); 4w: 59.8 (11.8)	12w:65 (33–94); 4w: 65 (26–93)	12w:58.6 (11.2); 4w: 59.2 (11.1)
Patient inclusion criteria	MBC (bone involvement) treated with ZOL every 3–4 weeks for 12–15 months before enrolment	MBC, prostate cancer, or multiple myeloma (bone involvement); PS score: 0–2; CC ≥ 30 mL/min; Ca: 2.00–2.90 mmol/L	MBC (bone involvement); had received bisphosphonate for ≥ 9 doses
Sample size	209 (12w); 216 (4w)	911 (12w); 911 (4w)	203 (12w); 200 (4w)
ZOL dosage per time	4 mg	nearly 4 mg ,but adjusted for calculated CC using actual body weight	4 mg
Supplementary medications	daily calcium (500 mg) and vitamin D (400–500 IUs)	daily calcium (500 mg) and vitamin D (400–800 IUs)	daily calcium (1000–2000 mg) and vitamin D (400–800 IUs)
Median follow-up	0.92 years	1.20 years	1.00 years
Primary endpoint	SMR	SRE rate	SRE rate
Secondary endpoints	SREs, time to first SRE, pain, use of analgesics, N-telopeptide of type I collagen concentration, and safety	SRE rate, pain scores, PS scores, SMR, C-terminal telopeptide levels, and safety	Time to first SRE and SMR

Abbreviations: ZOL: zoledronic acid; SMR: skeletal morbidity rate; SREs: skeletal-related events; 12w: 12 week; 4w:4 week; MBC: metastatic breast cancer; PS: performance status; CC: creatinine clearance; Ca: serum calcium;SD: standard deviation.

### Methodological quality of the included studies

**Figure 2:**
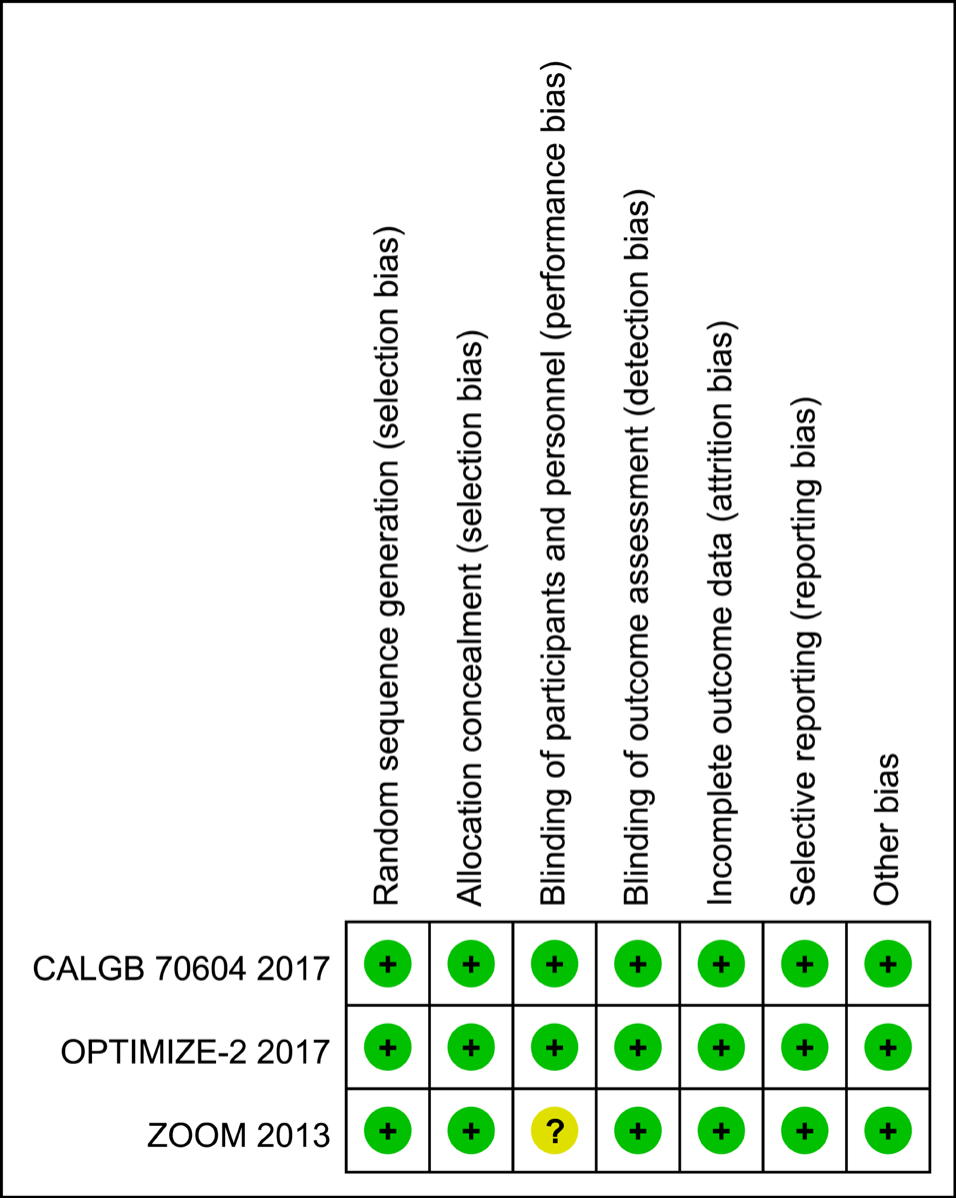
Summary of ‘Risk of bias’: reviewing authors’ judgments regarding risk of bias for every item in each of the included studies

The three included RCTs received a quality assessment: the baseline characteristics of the patients were reported in all RCTs, all studies mentioned “random” and reported an adequately randomized sequence generation, all trials reported methods of allocation concealment, and all reports described the reasons for incomplete outcome data. However, one study stated “Neither the patients nor the investigators were masked to treatment allocation”, which might have led to performance bias [13]. Figure 2 shows the qualities of the included trials.

### Skeletal-related events

**Figure 3:**
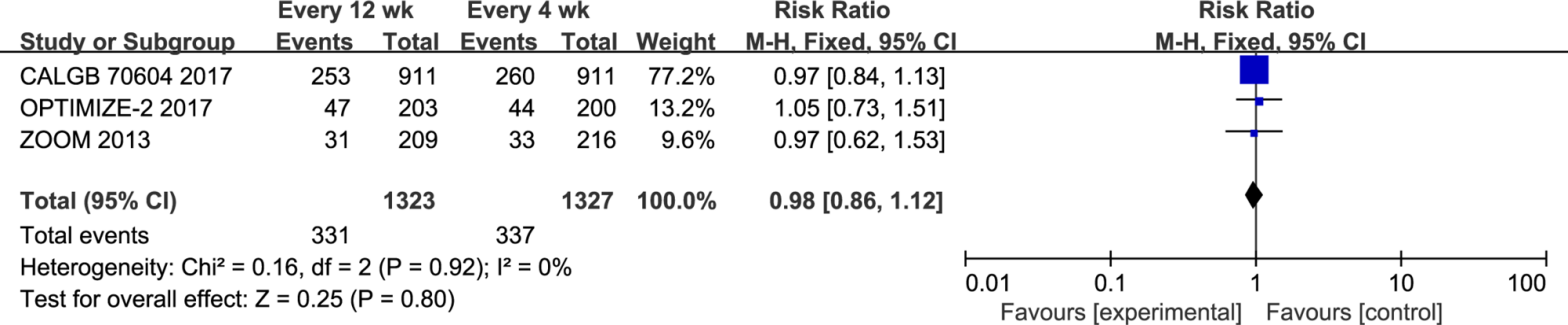
Forest plot of risk ratio for SREs

SREs were reported in all three studies (*n* = 2650). The fixed-effects model was used (chi^2^ = 0.16; *P* = 0.92; I^2^ = 0%), and no significant differences were observed between the 12-week and 4-week groups (RR = 0.98; 95% CI = 0.86–1.12; *P* = 0.80; Figure 3). The types of the SREs were the following: Radiation to bone, 208 patients in the zoledronic acid 12-week group, and 238 patients in the zoledronic acid 4-week group; pathological fractures, 107 patients in the 12-week group, and 86 patients in the 4-week group; spinal cord compression, 32 patients in the 12-week group and 24 patients in the 4-week group; surgery to bone, 44 patients in the 12-week group and 23 patients in the 4-week group; other types of SREs, 15 and 33 patients in the 12- and 4-week groups, respectively.

### Grade 3 or 4 adverse events

**Figure 4:**
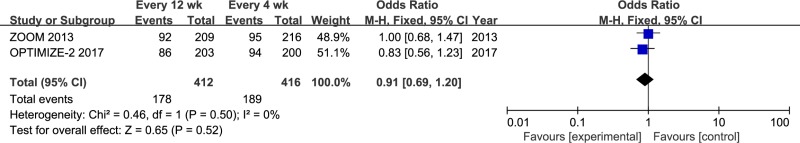
Forest plot of risk ratio for Grade 3 or 4 adverse events

Two studies (*n* = 828) reported grade 3 or 4 adverse events. The pooled estimate using the fixed-effect model indicated that the 12-week group did not experience a significant decrease in grade 3 or 4 adverse events (RR = 0.91; 95% CI = 0.69–1.20, *P* = 0.52; Figure 4).

### Osteonecrosis of the jaw

**Figure 5:**
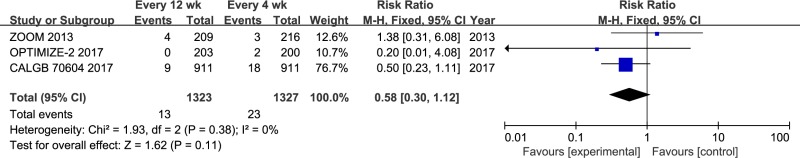
Forest plot of risk ratio for osteonecrosis of the jaw

Three studies (*n* = 2650) were included in the evaluation of osteonecrosis of the jaw. The results of our meta-analysis indicated that osteonecrosis of the jaw did not differ significantly between the 12-week and 4-week groups (RR = 0.58; 95% CI = 0.30–1.12; *P* = 0.11). In addition, the 12-week group had a lower incidence of jaw osteonecrosis [13 (0.98%) vs. 23(1.73%)]. No statistical heterogeneity was noted in this comparison (test for heterogeneity: I^2^ = 0%; *P* = 0.38; Figure 5).

### Kidney dysfunction

**Figure 6:**
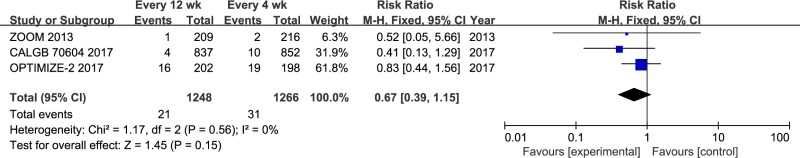
Forest plot of risk ratio for kidney dysfunction

All trials reported kidney dysfunction; 2514 patients were included in the meta-analysis. No significant differences were observed between the 12-week and 4-week groups, with low heterogeneity (RR = 0.67; 95% CI = 0.39–1.15; *P* = 0.15; *P* for heterogeneity = 0.56; I^2^ = 0%; Figure 6). However, the 12-week group had a decreased incidence of kidney dysfunction [21 (1.68%) vs. 31(2.45%)].

### Biomarkers of bone turnover

Three different turnover biomarkers were selected for the three studies, including N-terminal telopeptide [13], C-terminal telopeptide [11], and N-telopeptide to creatinine (uNTX:Cr) ratio [12]. Overall, the turnover biomarkers increased in 12-week groups in all three RCTs. In the 12-week group of the ZOOM study [13], N-terminal telopeptide concentration increased at 3 months and thereafter, while it did not change in the 4-week group. Similar results were seen in the longitudinal C-terminal telopeptide model of the CALGB70604 study [11], which found C-terminal telopeptide levels to be significantly higher in the 12-week group (*P* = 0.05). In addition, in the OPTIMIZE-2 study [12], the mean change from baseline profile of the uNTX:Cr ratio was comparable between the two treatment groups, except for one time point (36 weeks). However, due to the variety of turnover biomarkers in different studies, no statistical analysis was performed.

## DISCUSSION

Conventionally, zoledronic acid is administered every 3–4 weeks from the time of diagnosis of bone metastasis to death [20]. This dosage regimen was obtained from studies of patients with hypocalcemia who received anticancer agents [21]. However, these schedules failed to consider the toxicity associated with the long-term use of zoledronic acid [22]. Oncologists are increasingly interested in determining the optimal dosing interval that not only ensures the efficacy, but also reduces the toxicity of zoledronic acid [23].

In this meta-analysis of patients with bone metastases, 12-week dosing intervals of zoledronic acid were non-inferior compared to the standard 4-week dosing intervals in reducing the occurrence of SREs. This result is consistent with those of the three eligible clinical trials, indicating that the efficacy of the 12-week regimen is reliable. The most frequently recorded type of SREs in both treatment groups was radiation to bone, followed by pathologic fractures and spinal cord compression. Regarding the comparison of safety profiles, the grade 3 or 4 adverse events, osteonecrosis of the jaw, and kidney dysfunction were decreased, although no statistical differences were observed between the two schedules.

In 2015, a meta-analysis was reported on this topic [16], but that study examined bone-targeting agents, including pamidronate, zoledronate, and denosumab; data regarding zoledronic acid were not detailed. Therefore, those results were not sufficient to determine whether the intervals of zoledronic acid administration could be prolonged. To the best of our knowledge, this is the first meta-analysis to investigate whether zoledronic acid administration at 12-week intervals is suitable. Although our report only included three RCTs, they were of high quality. Moreover, the heterogeneity of the results was very low; hence, the results are credible.

Despite the lack of statistically significant differences in the efficacy and safety between the two schedules, the bone turnover biomarker concentrations (C-telopeptides and N-telopeptides) were higher in patients who received the 12-week regimen of zoledronic acid [11, 13]. Bone turnover biomarkers have been introduced in many studies as alternative indicators of mortality and subsequent SRE risk [24, 25]. The median follow-up times of the eligible RCTs in our study were one year only. It remains unclear whether these follow-up times were long enough to discover the differences in efficacy and safety between the two groups. Therefore, longer follow-up studies are warranted to ascertain whether SREs in the 12-week group increase with time. In addition, patients in the studied RCTs only included those with breast cancer, prostate cancer, and multiple myeloma [11–13]. Further study is required to investigate whether the results of our meta-analysis are applicable to other malignant tumors with bone metastasis, especially lung cancer.

In conclusion, our meta-analysis suggests that compared with the standard 4-week intervals, the administration of zoledronic acid at 12-week intervals does not lead to increased SREs, and may reduce the occurrence of jaw osteonecrosis and kidney dysfunction. This longer interval regimen may be an acceptable treatment choice. However, our results should be interpreted with caution, because the data are limited because of the insufficient patient population and short follow-up times. Larger RCTs with longer follow-up periods are needed to provide data applicable to clinical practice.

## SUPPLEMENTARY MATERIALS FIGURES AND TABLES



## Abbreviations

RR: risk ratio
CI: confidence interval
SREs: skeletal-related events
RCTs: randomized controlled trials
PRISMA: Systematic Reviews and Meta-Analyses
CTCAE: Common Terminology Criteria for Adverse Events
ZOL: Zoledronic acid
SMR: skeletal morbidity rate
12w: 12 weekly
4w: 4 weekly
MBC: Metastatic breast cancer
PS: performance status
Cc: creatinine clearance
Ca: serum calcium
SD: standard deviation
uNTX: CrN-telopeptide to creatinine
