# Correction: Systematic review and meta-analysis comparing zoledronic acid administered at 12-week and 4-week intervals in patients with bone metastasis

**DOI:** 10.18632/oncotarget.26009

**Published:** 2018-08-14

**Authors:** Ling Cao, Yong-Jing Yang, Jian-Dong Diao, Xu-He Zhang, Yan-Ling Liu, Bo-Yu Wang, Zhi-Wen Li, Shi-Xin Liu

**Affiliations:** ^1^ Department of Radiation Oncology, Cancer Hospital of Jilin Province, Changchun 130012, People’s Republic of China; ^2^ Department of Oncology and Hematology, China-Japan Union Hospital of Jilin University, Changchun 130012, People’s Republic of China; ^3^ Department of Anesthesiology, The First Hospital of Jilin University, Changchun, Jilin, 130021, People’s Republic of China

**This article has been corrected:** The correct affiliation is given below:

^2^ Department of Oncology and Hematology, China-Japan Union Hospital of Jilin University, Changchun 130012, People’s Republic of China

Original article: Oncotarget. 2017; 8:90308-90314. https://doi.org/10.18632/oncotarget.19856

