# Supplementary material for: Systematic review and meta-analysis comparing zoledronic acid administered at 12-week and 4-week intervals in patients with bone metastasis
Source: Oncotarget. 2017 Aug 3;8(52):90308–14. doi: 10.18632/oncotarget.19856 (PMC5685751; doi:10.18632/oncotarget.19856)
Supplement: Supplementary file 1 [file oncotarget-08-90308-s001.pdf]

Systematic review and meta-analysis comparing zoledronic acid administered at 12-week and 4-week intervals in patients with bone metastasis

SUPPLEMENTARY MATERIALS

Appendix: Pubmed search terms:

#1 ((((((((((((Zoledronic Acid[Title/Abstract]) OR 2-(imidazol-1-yl)-1-hydroxyethylidene-1, 1-bisphosphonic acid[Title/Abstract]) OR zoledronate[Title/Abstract]) OR Zometa[Title/Abstract]) OR Zometa[Title/Abstract]) OR Novartis brand of zoledronic acid[Title/Abstract]) OR CGP 42446A[Title/Abstract]) OR CGP-42446[Title/Abstract]) OR Aclasta[Title/Abstract]) OR Orazol[Title/Abstract]) OR Reclast[Title/Abstract])) OR «zoledronic acid» [Supplementary Concept]

#2 ((((((((Bone Neoplasm[Title/Abstract]) OR Bone Neoplasm[Title/Abstract]) OR Neoplasms, Bone[Title/Abstract]) OR Bone Cancer[Title/Abstract]) OR Cancer of the Bone[Title/Abstract]) OR Cancer of Bone[Title/Abstract]) OR bone[Title/Abstract])) OR "Bone Neoplasms"[Mesh]

#3 ((((((((((Metastases, Neoplasm[Title/Abstract]) OR Neoplasm Metastases[Title/Abstract]) OR Metastasis[Title/Abstract]) OR Metastases[Title/Abstract]) OR Metastasis, Neoplasm[Title/ Abstract]) OR Micrometastases [Title/Abstract]) OR micro-metastases[Title/Abstract]) OR micrometastasis[Title/Abstract]) OR micro-metastasis[Title/Abstract])) OR "Neoplasm Metastasis"[Mesh]

#4: (((((Randomized controlled trial[Title/Abstract]) OR controlled clinical trial[Title/Abstract]) OR randomized[Title/Abstract]) OR randomly[Title/Abstract]) OR trial[Title/Abstract]) OR random*[Title/Abstract]

#5: #1 AND #2 AND #3 AND #4
